# CHO cell lines generated by PiggyBac transposition

**DOI:** 10.1186/1753-6561-5-S8-P31

**Published:** 2011-11-22

**Authors:** Mattia Matasci, Virginie Bachmann, Lucia Baldi, David L Hacker, Maria De Jesus, Florian M Wurm

**Affiliations:** 1 Laboratory of Cellular Biotechnology, Faculty of Life Sciences, Ecole Polytechnique Fédérale de Lausanne, CH-1015 Lausanne, Switzerland; 2 ExcellGene SA, CH-1870 Monthey, Switzerland

## 

A major bottleneck in the manufacture of recombinant therapeutic proteins is the time and effort needed for the generation of stable, high-producing mammalian cell lines. Conventional gene transfer methods for stable cell line generation rely on random transgene integration, resulting in unpredictable and highly variable levels of expression of the transgene in individual clones [1,2]. As a consequence, a large number of stably transfected cells must be analyzed to recover a few high-producing clones. Recently, we described the use of a PiggyBac (PB) transposon for the generation of high-producing mammalian cell lines [3]. The PB dual vector system consists of 1) a donor vector carrying an artificial transposon with a mammalian expression cassette for the recombinant transgene and puromycin selection marker and 2) a helper vector driving transient expression of the PB transposase (PBase). PB transposition mediates stable transgene integration via a “cut and paste” mechanism in which the PBase excises the artificial transposon sequence from the plasmid and catalyzes its insertion into the host cell genome. A main advantages of the PB system over conventional passive integration are an improved efficiency of transgene integration resulting in a more integration events and more stable clones. Furthermore, PB favors transgene integration into actively transcribed regions of the host genome [4]. The PB transposon has a high cargo capacity of up to 14 Kb and transposition results in the stable genomic integration of well-defined sequences, thus reducing the probability of integration of truncated, non-functional transgenes [5]. Finally, recent reports have demonstrated the feasibility of using the PB system to obtain persistent expression of multiple genes carried either on a single or on distinct donor vectors [6].

**Figure 1:**
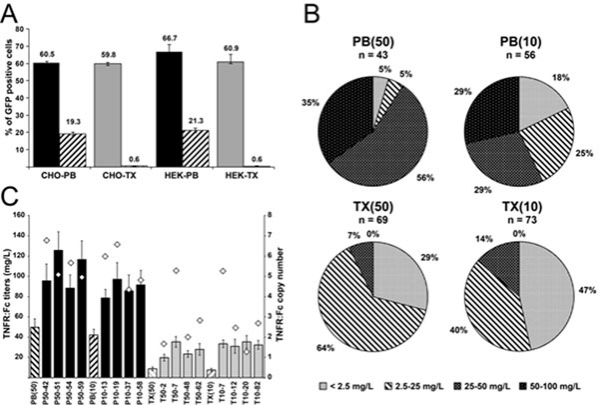
A) Enhanced stable integration efficiency in CHO and HEK 293 cells by PB transposition. B) Productivity analysis of CHO clonal cell lines sorted from cell pools generated by PB transposition (PB) or conventional transfection (TX). C) Analysis of the stability of TNFR:Fc expression over time and transgene copy number, for cell pools [PB(50), PB(10), TX(50), and TX(10)] and clonal lines generated by PB transposition (PB50 and PB10) or conventional transfection (TX50 and TX10).

We initially determined the efficiency of the PB system in the generation of stable lines using suspension adapted mammalian cells. CHO and HEK 293 cells were co-transfected with an eGFP-bearing donor plasmid along with the PB helper plasmid. The cells were then grown in the absence of puromycin, and the percentage of GFP-expressing cells in each culture was determined by flow cytometry on a daily basis. By 21 days post-transfection, the remaining GFP-positive cells were assumed to be recombinant. Compared to conventional transfection of plasmid DNA, PB transposition resulted in an improvement in the efficiency of stable cell line generation up to 20-fold for both CHO and HEK 293 cells (Figure 1A).

To further evaluate the PB system, CHO cells expressing a tumour necrosis factor receptor:Fc fusion protein (TNFR:Fc) were generated either by PB-transposition or by conventional transfection. Clonal cell lines were recovered following selection in 50 or 10 µg/mL puromycin for two weeks. Recovered lines were grown in suspension culture for 7 days in 24-well plates after which the medium was analyzed by ELISA to determine TNFR:Fc productivity. Transposition increased the frequency of high-producing clones in the transfected population (Figure 1B). To further characterized for the level and stability of transgene expression the original cell pools generated by PB transposition or conventional transfection, as well as the top 4 producers from each transfection were cultivated in the absence of selection in serum-free suspension culture, over a period of 16 or 14 weeks, respectively. When compared to clones and cell pools generated by conventional transfection, PB-derived cell lines and cell pools produced up to 4-fold more recombinant protein and had greater transgene expression stability (Figure 1C)

In conclusion our results demonstrate that stable cell lines derived by PB transposition are efficiently generated and are more productive than cell lines generated by conventional transfection methods. Therefore, the PB system represents a valuable and practical alternative to standard plasmid transfection to efficiently generate cell clones with stable and enhanced transgene expression.
